# Abnormal spontaneous brain activity in females with autism spectrum disorders

**DOI:** 10.3389/fnins.2023.1189087

**Published:** 2023-07-14

**Authors:** Jiapei Xie, Weidong Zhang, Yu Shen, Wei Wei, Yan Bai, Ge Zhang, Nan Meng, Xipeng Yue, Xinhui Wang, Xianchang Zhang, Meiyun Wang

**Affiliations:** ^1^Department of Medical Imaging, Zhengzhou University People’s Hospital and Henan Provincial People’s Hospital, Zhengzhou, China; ^2^Academy of Medical Sciences, Zhengzhou University, Zhengzhou, China; ^3^MR Collaboration, Siemens Healthineers Ltd., Beijing, China; ^4^Laboratory of Brain Science and Brain-Like Intelligence Technology, Institute for Integrated Medical Science and Engineering, Henan Academy of Sciences, Zhengzhou, China

**Keywords:** autism spectrum disorders, amplitude of low-frequency fluctuation, fractional amplitude of low-frequency fluctuation, regional homogeneity, spontaneous brain activity

## Abstract

**Objectives:**

To date, most studies on autism spectrum disorder (ASD) have focused on sample sets that were primarily or entirely composed of males; brain spontaneous activity changes in females remain unclear. The purpose of this study was to explore changes in the brain spontaneous neural activity in females with ASD.

**Methods:**

In this study, resting-state functional magnetic resonance images (rs-fMRI) of 41 females with ASD and 41 typically developing (TD) controls were obtained from the ABDIE database. The amplitude of low-frequency fluctuation (ALFF), fractional ALFF (fALFF), and regional homogeneity (ReHo) of the two groups were calculated to detect the regional brain activity. A two independent sample *t*-test was used to analyze differences between the ASD and TD groups and a *p-*value <0.05 was considered statistically significant after false discovery rate (FDR) correction. Pearson correlation analysis was conducted between social responsiveness scale (SRS) scores and the local activity of significantly different brain regions.

**Results:**

Compared with the typically developing (TD) group, the values of ALFF and ReHo were significantly increased in the left superior temporal gyrus (STG), while the values of ReHo were significantly decreased in the left superior frontal gyrus (SFG), left middle occipital gyrus (MOG), bilateral superior parietal lobule (SPL), and bilateral precuneus in the females with ASD group. Correlation analysis showed that the ReHo of the right precuneus was positively correlated to the total SRS, social communication, and autistic mannerisms.

**Conclusion:**

Spontaneous activity changes in females with ASD involved multiple brain regions and were related to clinical characteristics. Our results may provide some help for further exploring the neurobiological mechanism of females with ASD.

## Introduction

Autism spectrum disorder (ASD) is a neurodevelopmental disorder that is highly heritable heterogeneous, and is characterized by impairment of social interaction and communication, repetitive or stereotyped behavior, and restricted interests (Philip et al., 2012). The estimated prevalence of ASD stands at approximately 1 in 54 children, with a male to female ratio of 4.3:1 (Matthew et al., 2016). Patients with ASD have poor self-care, which places a huge burden on families and society. However, the exact pathophysiological mechanism of ASD is still poorly understood; thus, early diagnosis and effective treatment of ASD need to be further explored.

The resting-state is defined as a state in which a subject does not perform any task. In this state, most energy is provided to the brain spontaneous activity, which causes changes in blood flow and blood oxygen levels in local brain regions (Lv et al., 2018; Raimondo et al., 2021). Functional magnetic resonance imaging (fMRI) enables detection of changes in the brain, defined as blood oxygen level–dependent (bold) signals (Lee et al., 2013). Regional homogeneity (ReHo), based on Kendall’s coefficient concordance (KCC), is used to measure the similarity of a time series between a given voxel and its nearest neighbors (Zang et al., 2004). Amplitude of low-frequency fluctuation (ALFF) measures the amplitude of fluctuation in the time series of each voxel in the range 0.01–0.08 Hz and fractional ALFF (fALFF) measures the relative contribution of low-frequency fluctuations to the entire range of detectable frequencies (Zang et al., 2007; Zou et al., 2008). Compared with functional connectivity (FC), which reveals temporal correlations between brain regions, ReHo, ALFF, and fALFF do not require *a priori* assumptions to determine the seed region. At the same time, abnormal brain regions determined from the results of ReHo, ALFF, and fALFF can be used as seeds for FC analysis. ReHo, ALFF, and fALFF values were used to evaluate spontaneous brain activity and have been successfully applied in the study of various neurological and mental disorders such as attention-deficit/hyperactivity disorder (Shang et al., 2016, 2021), Alzheimer’s disease (Song et al., 2021), schizophrenia (Sun et al., 2021), and Parkinson’s disease(Yue et al., 2020).

fMRI showed that the neural mechanism was associated with abnormal brain function in ASD (Karavallil Achuthan et al., 2023). However, most studies to date have focused on sample sets that were primarily or entirely composed of males; the neural activity traits of females remain unclear. Previous studies found that the clinical manifestations were different between males and females (Green et al., 2019). Compared with males with ASD, females with ASD have (1) less restricted, repetitive, and stereotyped behavior (Hartley and Sikora, 2009); (2) more camouflage to hide their symptoms (Lawrence et al., 2020); (3) more psychiatric co-morbidity conditions such as depressive disorders, anxiety disorders, and attention-deficit/hyperactivity disorder (ADHD) (Hartley and Sikora, 2009); (4) more empathy (Harmsen, 2019); and (5) more social challenges and friendship conflicts (Sedgewick et al., 2019). Although females with ASD account for very small proportion of people with ASD, due to the high prevalence of autism, they should not be ignored and deserve to be further studied. In addition, females with ASD tend to hide their emotional and social disorders, and thus they are more likely to be misdiagnosed or have a delayed diagnosis. This study explores the abnormal brain activity of females with ASD and provides objective evidence for its early diagnosis.

The values of ReHo, ALFF, and fALFF have previously been used to explore brain spontaneous activity changes in ASD; however, no studies have been published on that in females. In this study, we performed ReHo, ALFF, and fALFF analyses on females with ASD from the Autism Exchange database based on resting-state fMRI (rs-fMRI). We aimed to explore the brain spontaneous activity of females with ASD, which will contribute to understanding the neural mechanism and will have important clinical significance for timely diagnosis and intervention.

## Materials and methods

### Participants

fMRI data of ASD groups and typical development (TD) groups were obtained from the autism brain image data exchange (ABIDE) project. In our study, we included seven collection sites (ABIDEII-GU, ABIDEII-KKI, ABIDEII-NYU_1, ABIDEII-NYU_2, ABIDEII-OHSU, ABIDEII-SDSU, and ABIDEII-UCD). According to the ethics board policies, our study was exempt from ethical review. The acquisition parameters, informed consent, diagnostic criteria, and specific protocols of each site are available on the database website.^1^ ASD groups were determined according to the following criteria: (1) female patients; (2) subjects with complete fMRI and structural imaging, (3) subjects with low head motion (maximum translation <3 mm or maximum rotation <3° in all three directions), and (4) the social responsiveness scale (SRS) was available. In total, 41 patients with ASD and 41 age- and sex-matched TDs were included in our study. SRS is a widely used measure of autism symptoms. It provides a total scale and five subscales including total, social awareness, social cognition, social communication, social motivation, and autistic mannerisms. Higher SRS scores indicate a higher severity of ASD clinical symptoms (Ma et al., 2021).

### Data preprocessing

Matlab R2016b^2^ and RESTplus V1.25^3^ data analysis toolkits (Jia et al., 2019) were used for image preprocessing and analysis. The preprocessing process was as follows: (1) the first 10 time points were removed to eliminate the instability of the initial MRI signal; (2) time correction and realignment were conducted to exclude participants with excessive head motion (maximum translation >3 mm or maximum rotation >3° in all three directions); (3) fMRI data were co-registered to the T1 image to align them in the same spatial space. The T1 image was segmented into tissue probability maps and normalized to the Montreal Neurological Institute (MNI) standard template. The corresponding transformation matrices from the registration process were then applied to the fMRI data, warping them into the standard template space, with both images resampled to 3 mm isotropic voxels; (4) a Gaussian kernel of 6 mm full-width at half-maximum (FWHM) was used for spatial smoothing before ALFF and fALFF calculation; (5) linear regression was used to reduce the influence of the MRI equipment; (6) nuisance covariates regression analysis was performed with friston-24 head motion, cerebrospinal fluid (CSF), and white matter signals, aiming to reduce the influence of head motion and non-neuronal BOLD fluctuations; and (7) time-domain bandpass filtering (0.01–0.08 Hz) was performed to reduce the effects of low-frequency drift and high-frequency noise before ReHo calculation.

### ALFF, fALFF, and ReHo analyses

RESTplus V1.25 was used to calculate the values of ALFF, fALFF, and ReHo (Jia et al., 2019). After data preprocessing, the ALFF and fALFF values were calculated according to the following steps: we used fast Fourier transform to transform the time series of each voxel into the frequency domain and calculated the power spectrum of each voxel. ALFF values were calculated by taking the averaged square root of each frequency of the power spectrum at each voxel across 0.01–0.08 Hz (Zang et al., 2007). fALFF values were calculated as the ratio of the power spectrum of the low-frequency range (0.01–0.08 Hz) to that of the entire frequency range (Zou et al., 2008). Raw ALFF and fALFF values were converted to *Z*-scores for group comparison. The ReHo values were calculated according to the following steps: the similarity between a single voxel and the surrounding 27 voxels was determined according to Kendall’s coordination coefficient (KCC). For purposes of standardization, the ReHo values of each voxel were converted to a *Z*-score. Finally, spatial smoothing was performed using a 6 mm smoothing kernel.

### Statistical analyses

Statistical product and service solutions (SPSS 22.0, IBM, Armonk, NY, United States) was used for statistical analyses. A two-sample test was used to compare differences in the age of the groups. Differences in ALFF, fALFF, and ReHo between the ASD and TD groups were calculated by a two-sample test based on the data analysis toolkit RESTplus V1.25 and a *p-*value <0.05 was considered statistically significant after false discovery rate (FDR) correction. The ALFF, fALFF, and ReHo values were extracted from significantly different brain regions and Pearson correlation analysis was conducted on the scores of the SRS scales (*p* < 0.05).

## Results

### Participants

The average age of the ASD and TD groups was 10.98 ± 3.08 and 10.71 ± 2.01, respectively, and there was no significant difference between the two groups (*t* = −0.480, *p* = 0.633). The SRS total scale and the five subscale scores of the ASD group were obtained from the database. The demographics and clinical characteristics arere listed in Table 1.

**Table 1:** Demographic and clinical characteristics of females with ASD and the TD group.

	ASD group (*n* = 41)	TD group (*n* = 41)	*T*	*P*
Age	10.98 ± 3.08	10.71 ± 2.01	−0.480	0.633
SRS_TOTAL	96.24 ± 30.40			
SRS_AWARENESS	12.41 ± 4.42			
SRS_COGNITION	17.83 ± 5.94			
SRS_COMMUNICATION	32.73 ± 11.11			
SRS_MOTIVATION	15.34 ± 5.79			
SRS_MANNERISMS	17.93 ± 7.07			

### Aberrant local activity

**Figure 1:**
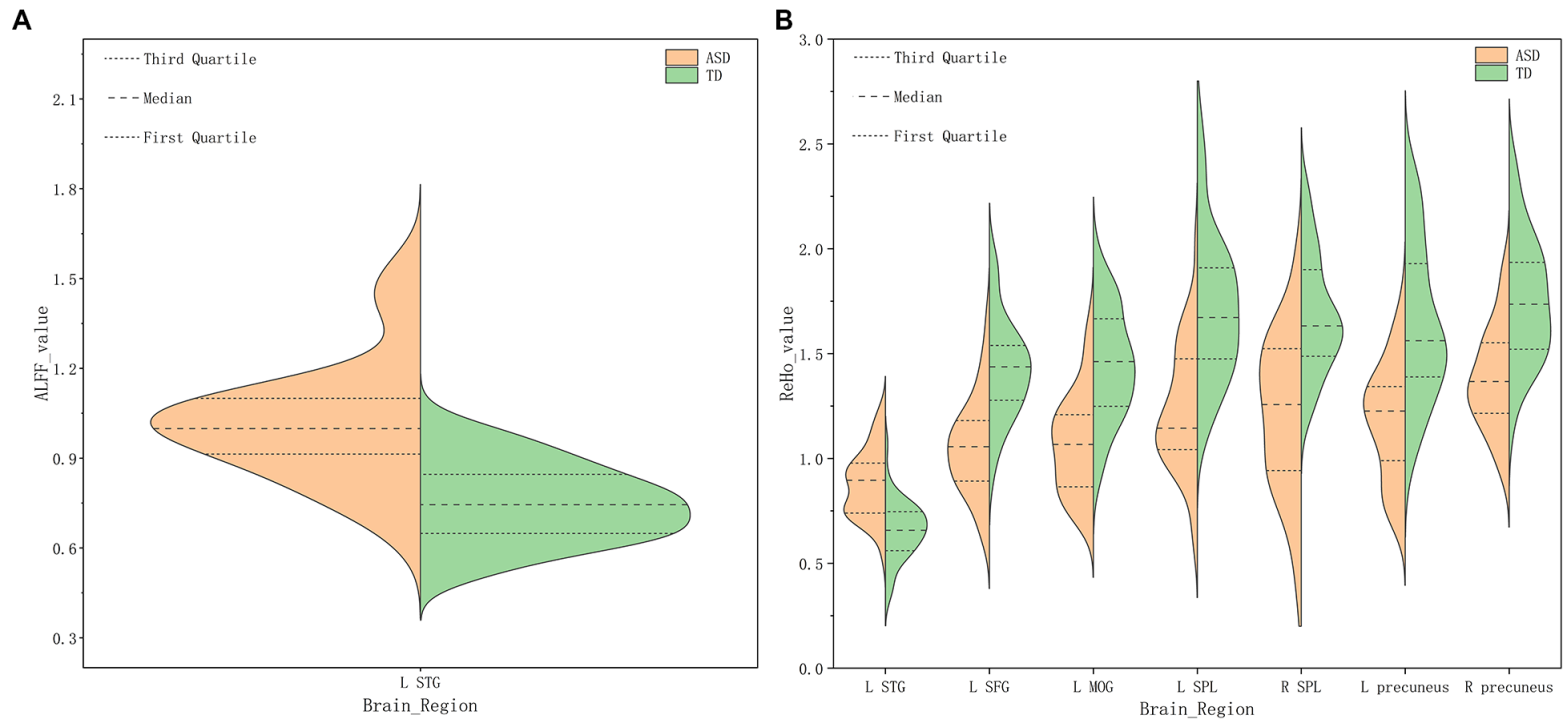
Amplitude of low-frequency fluctuation (ALFF) **(A)**, Regional homogeneity (ReHo) **(B)** values for altered regional brain regions in ASD and TD females.

**Figure 2:**
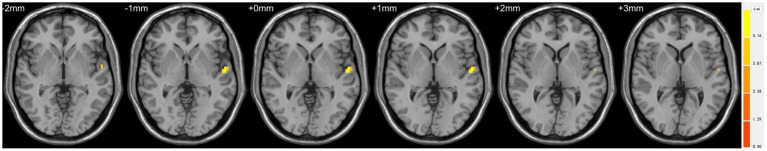
Statistically significant differences in the left superior temporal gyrus between females in the ASD and TD groups. Yellow colors denote increased ALFF values. The color bars indicate the *t* value.

**Figure 3:**
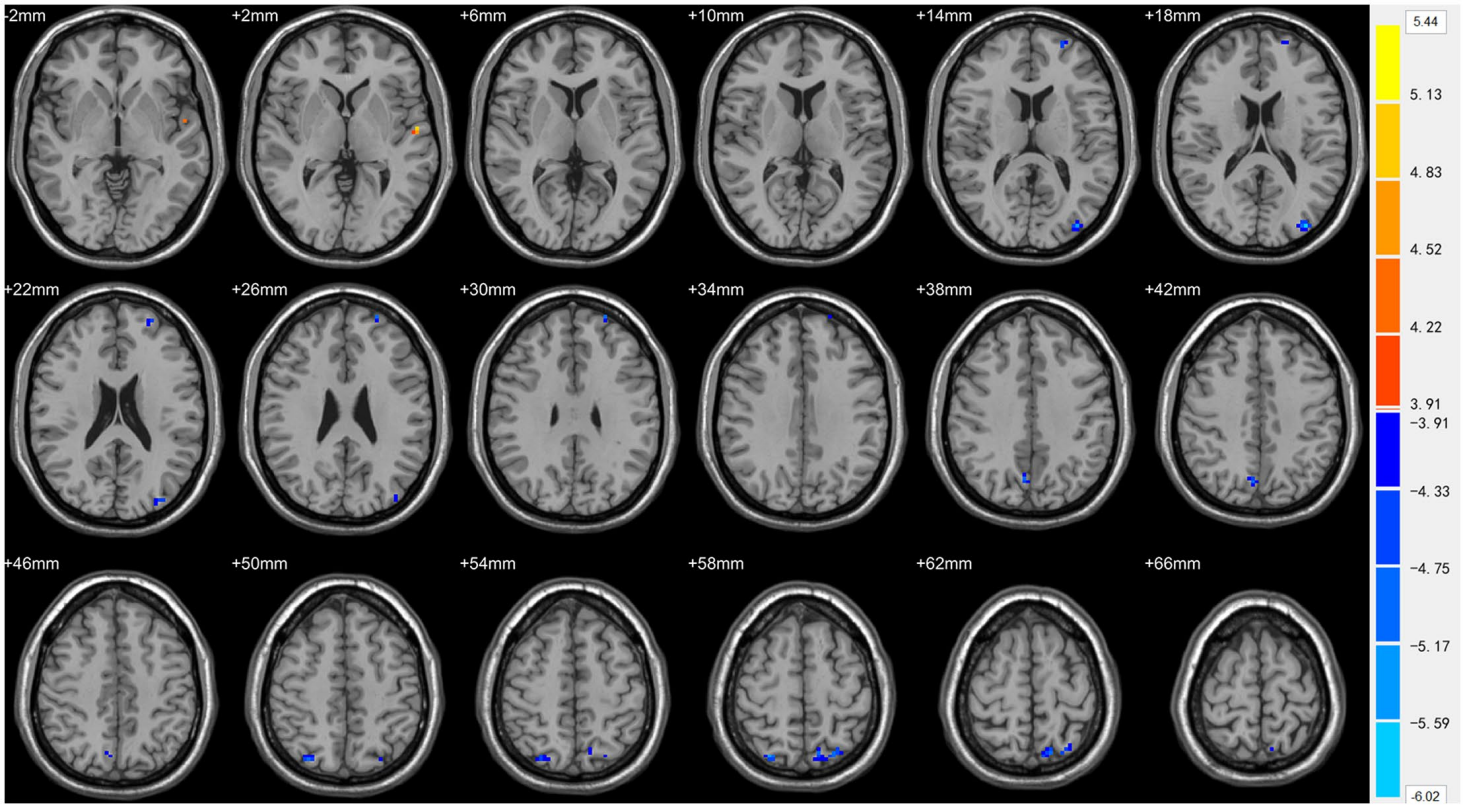
Statistically significant differences in the left superior temporal gyrus, left superior frontal gyrus, left middle occipital gyrus, bilateral superior parietal lobule, and bilateral precuneus between females in the ASD and TD groups. Orange yellow colors denote increased ReHo values; blue colors denote decreased ReHo values. The color bar indicates the *t* value.

Compared with TDs, we found abnormal changes in several brain regions of females with ASD. The results showed significantly increased ALFF and ReHo values in the left superior temporal gyrus (STG) and decreased ReHo values in the left superior frontal gyrus (SFG), left middle occipital gyrus (MOG), bilateral superior parietal lobule (SPL), and bilateral precuneus (Table 2 and Figures 1–3).

**Table 2:** Brain regions with abnormal ALFF and ReHo values in females with ASD.

	Brain region	Cluster size	MNI coordinates			AAL	Peak *T*-value
			*X*	*Y*	*Z*		
ALFF	L STG	10	−57	−9	0	Temporal_Sup_L	6.4556
ReHo	L STG	10	−57	−9	0	Temporal_Sup_L	5.4397
	L SFG	16	−24	57	12	Frontal_Sup_L	−5.1896
	L MOG	23	−36	−87	18	Occipital_Mid_L	−6.016
	L SPL	20	−24	−69	60	Parietal_Sup_L	−5.573
	R SPL	18	27	−72	57	Parietal_Sup_R	−5.3477
	L precuneus	16	−12	−69	60	Precuneus_L	−4.929
	R precuneus	14	6	−72	42	Precuneus_R	−5.1162

L, left; R, right; ALFF, amplitude of low-frequency fluctuation; ReHo, regional homogeneity; STG, superior temporal gyrus; SFG, superior frontal gyrus; MOG, middle occipital gyrus; SPL, superior parietal lobule; MNI, Montreal Neurological Institute; AAL, anatomical automatic labeling.

### Correlation between local activity and autism traits

**Figure 4:**
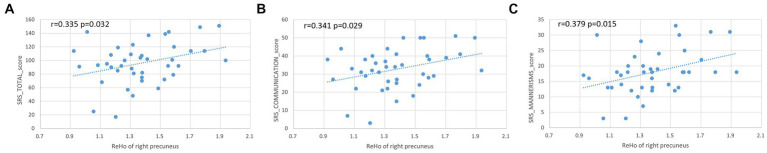
Positive correlation between ReHo values of right precuneus with SRS total, social communication, and autistic mannerisms **(A–C)**.

The ReHo value of the right precuneus was positively correlated to the SRS total (*r* = 0.335, *p* = 0.032), social communication (*r* = 0.341, *p* = 0.029), and autistic mannerisms (*r* = 0.379, *p* = 0.015, Figure 4).

## Discussion

Based on resting-state functional magnetic resonance imaging (rs-fMRI), ALFF, fALFF, and ReHo values were analyzed to explore spontaneous brain activity changes in females with ASD. Compared with TDs, we found abnormal changes in several brain regions of females with ASD. The results showed significantly increased ALFF and ReHo values in the left STG, and decreased ReHo values in left SFG, left MOG, bilateral SPL, and bilateral precuneus. The ReHo values of the right precuneus were positively correlated to the SRS total, social communication, and autistic mannerisms.

The frontal lobe has been associated with a wide range of cognitive control functions. The SFG is involved in a variety of functions and is composed of several functional subregions. Li et al. (2013) divided the SFG into anteromedial (SFGam), dorsolateral (SFGdl), and posterior (SFGp) subregions and suggested that the SFG was responsible for self-referential processing, cognitive functions, execution, and motor control. We found that the ReHo values of females with ASD were decreased in the left SFG. The results were similar to a study of males with ASD, in which Shukla et al. showed reduced ReHo values in the bilateral SFG (Shukla et al., 2010). The SFG is thought to contribute to many essential higher functions. It is not surprising that the SFG was disrupted in both males and females with ASD. We believe that dysfunction of the SFG is closely related to the clinical symptoms of autism, which seriously affect cognition, execution, and motor control.

The STG contains the non-primary auditory cortex and plays a critical role in hearing, perception, understanding auditory and visual speech information, and vocal expressions of emotions (Fruhholz and Grandjean, 2013; Anderson et al., 2017; Yi et al., 2019; Bhaya-Grossman and Chang, 2022). Hearing and speech are prerequisites for social interaction. A previous study found that females with ASD have more social challenges and more friendship conflicts, which was related to inflexible language comprehension and declining emotion perception (Sedgewick et al., 2019). In our study, females with ASD exhibited increased ALFF and ReHo values in the left STG compared to the TD group. Dajani and Uddin (2016) also observed higher ReHo values in the STG of children with ASD. This may be related to social impairment in ASD. A previous study suggested that empathy was significantly associated with STG activity in ASD (Kana et al., 2016). Increased ReHo values in the STG may explain the stronger empathizing ability of females with ASD (Harmsen, 2019). In addition, the STG was also associated with cognitive performance and implicit emotion processing (Achiron et al., 2013; Kana et al., 2016). Therefore, the disruption of STG local activity has important clinical implications in the neural mechanisms of females with ASD.

The precuneus, a part of the medial posterior parietal cortex, plays a central role in a wide range of cognitive processes and highly integrated tasks, including spatially guided behaviors, mental imagery, episodic memory retrieval, and self-processing operations, with the ventral precuneus being part of the default mode network (Cavanna and Trimble, 2006; Zhang and Li, 2012). Precuneus dysfunction in autistic individuals has been frequently reported in previous studies. Li et al. (2018) indicated that boys with ASD (mean age: 8.87 ± 3.11) exhibited increased local functional homogeneity in the right precuneus. Shukla et al. (2010) found that the ReHo values in the right precuneus were lower in the ASD group (11–18 years) than in the TD group. Guo et al. (2017) identified significantly reduced ALFF values in the right precuneus across adolescents and adults with ASD. Thus, the brain function changes of the precuneus may be closely related to the developmental stage, increasing before puberty and gradually decreasing after. We mainly included children and adolescents (5.2–18 years) who showed decreased ReHo values in the bilateral precuneus. This demonstrates the global characteristics of local functional alterations in the precuneus in females with ASD. In addition, we found that the ReHo values of the right precuneus were positively correlated to the SRS total, social communication, and autistic mannerisms, which indicated that atypical changes in the precuneus were closely related to social interaction impairment and repetitive or stereotyped behavior. This is consistent with the previous study by Guo et al. (2017), who suggest that precuneus abnormalities may contribute to social and emotional disorders. Thus, we speculate that the local activity of the precuneus is an effective imaging marker for evaluating the severity of ASD in females.

The superior parietal region is part of the dorsal attention network (DAN), which is implicated in the top-down allocation of attentional resources and related to three cognitive functions (working memory, episodic retrieval, and mental imagery) (Luckmann et al., 2014; Cona et al., 2017). Newman et al. (2003) found left/right differences in the superior parietal region and revealed that the right side is more involved in attention processes and that the left side is more responsible for the visuo-spatial workspace. We found decreased ReHo values in the bilateral SPL in females with ASD. Shukla et al. also found lower ReHo values in the left SPL (Shukla et al., 2010). These results indicate a relationship between the impaired cognitive functions of females with ASD and an abnormal SPL. A large number of studies found abnormal structure and functionality in the SPL, including parietal cortical thinning, decreased local activity, and reduced cerebral blood flow (Wallace et al., 2010; Ye et al., 2022). In addition, the SPL has an important influence on motor learning and repetitive behavior in ASD. Travers et al. (2015) suggested that activation of the SPL was decreased during motor learning and was strongly associated with more severe, repetitive behavior/restricted interest symptoms.

The MOG, a part of the occipital lobe, is responsible for the processing of visual information and the perception of facial emotion (Teng et al., 2018). Harrop et al. (2019) suggested that females with ASD afforded less visual attention to faces than the TD group. During a face perception task, activation of the occipital gyrus was significantly reduced in participants with ASD compared to normal control subjects (Pierce et al., 2001). In our study, the ReHo values were reduced in the left MOG in females with ASD compared with females in the TD group, which indicated that the local activity of MOG was decreased. Similar to our results, Itahashi et al. (2015) reported that adults with high-functioning ASD showed significantly decreased fALFF values in the right MOG. Guo et al. (2017) found that the ALFF values were decreased in the left MOG during all developmental stages in people with ASD, and this could predict the ADOS social subscore. Therefore, abnormal local activity of the MOG holds great value for evaluating social impairment in females with ASD.

In conclusion, abnormal brain regions in females with ASD are generally similar to those in males with ASD, which reflects the commonality of brain abnormalities across the ASD population. However, whether there are gender differences in these abnormal brain regions needs to be further studied. In addition, many studies have reported that males with ASD have abnormal changes in the postcentral gyrus (Paakki et al., 2010; Zhao et al., 2022), precentral gyrus (Dajani and Uddin, 2016; Zhao et al., 2022), middle frontal gyrus (Paakki et al., 2010; Shukla et al., 2010; Di Martino et al., 2014), middle temporal gyrus (Shukla et al., 2010; Li et al., 2018; Lan et al., 2021), and right superior temporal sulcus (Paakki et al., 2010; Jiang et al., 2015). However, no difference was found in our female patients, which may be due to gender differences. Therefore, focusing on females and exploring the brain activity characteristics in females will help us to deepen understanding of the neural mechanism of females with ASD and to provide timely and targeted interventions.

Clinical characteristics, including sensorimotor, cognitive, and socio-communicative disorder in ASD, were closely related to abnormalities in functional and anatomical connectivity (Maximo et al., 2013). Our study investigated the relationship between local activity and clinical traits of females with ASD. However, there are still some limitations to our study. First, our sample size is relatively small. Since the number of male patients is 4.3 times higher than female patients, and because females are more difficult to recruit, we need more female patients in order to prove the results of our study in the future. Second, this is a cross-sectional study. Atypical brain development trajectories have been proved in ASD. Future longitudinal studies in females with ASD are needed. Finally, the study only included females with ASD and thus the conclusions only apply to females with ASD.

## Conclusion

Based on rs-fMRI data, females with ASD showed abnormal brain function in the left SFG, left STG, left MOG, bilateral SPL, and bilateral precuneus, which mainly showed decreased spontaneous brain activity. The ReHo values of the right precuneus were positively correlated to the SRS total, social communication, and autistic mannerisms. Our results provide evidence for understanding the neural mechanisms of females with ASD and have important clinical significance for the early diagnosis and precision treatment of different symptoms.

## Data availability statement

The original contributions presented in the study are publicly available. This data can be found here: http://fcon_1000.projects.nitrc.org/indi/abide/abide_II.html.

## Ethics statement

The data were obtained from public databases. According to the institutional ethical review board policies, ethical review was waived for this research.

## Author contributions

JX: validation, formal analysis, investigation, and writing – original draft. WZ: methodology. YS: software. WW, YB, and GZ: validation. NM, XY, XW, and XZ: data curation. MW: formal analysis, data curation, and writing – review and editing. All authors contributed to the article and approved the submitted version.

## Funding

This study has received funding by the National Natural Science Foundation of China (81720108021).

## Conflict of interest

XZ were employed by Siemens Healthineers Ltd.

The remaining authors declare that the research was conducted in the absence of any commercial or financial relationships that could be construed as a potential conflict of interest.

## Publisher’s note

All claims expressed in this article are solely those of the authors and do not necessarily represent those of their affiliated organizations, or those of the publisher, the editors and the reviewers. Any product that may be evaluated in this article, or claim that may be made by its manufacturer, is not guaranteed or endorsed by the publisher.

## Footnotes

^1^ http://fcon_1000.projects.nitrc.org/indi/abide/

^2^ https://ww2.mathworks.cn/products/matlab.html

^3^ http://www.restfmri.net/forum/
